# Graft Infections in Biologic Reconstructions in the Oncologic Setting: A Systematic Review of the Literature

**DOI:** 10.3390/jcm13164656

**Published:** 2024-08-08

**Authors:** Andrea Sambri, Renato Zunarelli, Lorenzo Morante, Chiara Paganelli, Stefania Claudia Parisi, Marta Bortoli, Andrea Montanari, Michele Fiore, Cristina Scollo, Alessandro Bruschi, Massimiliano De Paolis

**Affiliations:** Orthopedic and Traumatology Unit, IRCCS Azienda Ospedaliero-Universitaria di Bologna, 40138 Bologna, Italy; renato.zunarelli@ior.it (R.Z.); lorenzo.morante@ior.it (L.M.); chiara.paganelli8@studio.unibo.it (C.P.); stefania.parisi@ior.it (S.C.P.); marta.bortoli@ior.it (M.B.); andrea.montanari36@studio.unibo.it (A.M.); michele.fiore@aosp.bo.it (M.F.); cristina.scollo@aosp.bo.it (C.S.); alessandro.bruschi@aosp.bo.it (A.B.); massimiliano.depaolis@aosp.bo.it (M.D.P.)

**Keywords:** infections, allografts, vascularized fibula, failure, oncologic

## Abstract

**Background:** Biologic graft infection (BGI) is one of the main complications in graft reconstructions. However, very little evidence exists regarding the epidemiology of BGI, as most of the data come from sparse reports. Moreover, most of the series did not detail the treatment and outcome of graft infections. The aim of this systematic review of the literature is to provide a comprehensive data collection on BGI after oncologic resections. **Methods:** Three different databases (PubMed, Scopus, and Web of Science) were searched for relevant articles, and further references were obtained by cross-referencing. **Results:** 139 studies met the inclusion criteria. A total of 9824 grafts were retrieved. Among these, 684 (6.9%) were in the humerus, 365 (3.7%) in the pelvis, 2041 (20.7%) in the femur and 1660 (16.8%) in the tibia. Most grafts were osteoarticular (2481, 26.7%) and intercalary 2112 (22.7%) allografts. In 461 (5.0%), vascularized fibula grafts (VFGs) were used in combination with recycled autografts. Recycled grafts were reported in 1573 (16.9%) of the cases, and allograft-prosthetic composites in 1673 (18.0%). The pelvis and the tibia had the highest incidence of BGI (20.4% and 11.0%, respectively). The most reported first treatment was debridement and implant retention (DAIR) in 187 (42.8%) cases and two-stage revision with graft removal in 152 (34.8%). Very little data are reported on the final outcome specified by site or type of graft. **Conclusions:** This systematic review of the literature confirms a high incidence of infections in biologic reconstructions after resections of primary bone tumors. Despite DAIR being a viable attempt, in most cases, a two-stage approach with graft removal and reconstruction with endoprosthesis presented the highest chance to overcome infection, guaranteeing a reconstruction. We emphasize the need for future multicentric studies to focus on the management of infections after biological reconstructions in bone sarcomas.

## 1. Introduction

Massive bone grafts have been used for several decades in orthopedic oncology [1]. The most popular biological reconstructions after segmental resection of a bone sarcoma include allografts [2,3,4], vascularized fibula graft (VFG) [5,6], combined allograft and VFG [7,8,9], extracorporeal devitalized (recycled) autograft [10], and massive grafts in combination with a prosthesis (allograft-prosthetic composite, APC) [11].

Bone allografts have many advantages, as they allow adequate attachment of salvaged tendons and provide initial mechanical strength [12]. The graft is progressively incorporated by the host during healing through creeping substitution and may survive for decades. Moreover, it is possible to preserve host bone stock without donor site morbidity [13,14] either in osteoarticular or intercalary diaphyseal bone defects.

On the other hand, vascularized bone grafts do not undergo resorption by creeping substitution due to their intact vascularity, but they require microvascular reconstruction, which requires prolonged surgical procedures and surgical expertise, difficult to be easily reproducible. VFG can be used in three forms of reconstructive options: 1. single vascularized fibular graft, which is mainly indicated for reconstruction of areas with lower mechanical load as in upper extremity reconstruction, segmental defects of the mid-tibia, and intercalary defects in pediatric patients; 2. vascularized double-barreled fibula, which can be indicated for areas with intermediate mechanical stress such as femur and pelvis; and 3. in combination with an allograft or recycled autograft [6,7]. However, donor site morbidity is high, including bleeding, peroneal nerve palsy, contracture of flexor hallucis longus, and ankle pain [15].

Contrarily, allograft-prosthetic composites present several advantages to reconstructing articular segments: they allow for a biologic restoration of the bone defect, they share the load with the prosthesis once bone union is achieved, and they allow soft tissue attachments around the reconstructed joint [11]. However, the results of APCs seem to vary greatly, given the anatomic site involved.

Additionally, biological reconstruction performed via reuse of the devitalized resected tumor-bearing bone is common in certain Asian countries as the concept of bone donation is not widely accepted. The basic idea behind implanting devitalized (recycled) bone is to reimplant the resected bone after extracorporeal devitalization of the tumor. [16,17]. The techniques of devitalizing procedures include irradiation, autoclaving, pasteurization, or freezing with liquid nitrogen. Reconstruction is performed by reimplantation of the devitalized autograft and stabilization by suitable osteosynthesis [18,19].

In general, biologic graft infection (BGI) is one of the main complications in graft reconstructions, usually resulting in early failure of the reconstruction [1]. Innocenti et al. [20] suggested that VFG is more resistant to infection (owing to its vascularity). However, very little evidence exists regarding the epidemiology of BGI, as most of the data come from sparse reports. Moreover, most of the series did not detail the treatment and outcome of graft infections.

The aim of this systematic review of the literature is to provide a comprehensive data collection on infections of graft reconstructions after oncologic resections and to provide an overview of this topic, especially from an epidemiologic point of view.

## 2. Materials and Methods

This systematic review was conducted in accordance with the 2020 PRISMA guidelines (Preferred Reporting Items of Systematic Reviews) [21].

All studies (randomized controlled trials (RCT), prospective (PCCS) and retrospective comparative studies (RCCS), prospective (PCS) and retrospective case series (RCS)) reporting on deep infection cases of graft reconstructions were included. Biomechanical studies, cadaveric studies, “in vitro” studies, and animal model studies were excluded. Only articles written in English and published in a peer-reviewed journal were included. Articles published prior to 1985 were also excluded.

The criteria used to select articles allowed for the extrapolation of data about infections of graft reconstructions. Studies eligible for this systematic review were identified through an electronic systematic search of PubMed, Scopus, and Web of Science until 31 July 2024. The search string used was as follows: (graft OR allograft OR autograft OR vascularized fibula) AND (sarcoma OR bone tumour) AND (infection OR deep infection OR failure OR complication). Articles without an abstract were excluded from the study. Screening of the articles was conducted by considering the relevance of titles and abstracts and looking for the full-text article when the abstract provided insufficient information about inclusion and exclusion criteria.

Articles that were considered relevant by electronic search were retrieved in full text, and a cross-referencing search of their bibliographies was performed to find further related articles. Reviews and meta-analyses were also analyzed in order to broaden the search to studies that might have been missed through the electronic search. All duplicates were removed, and all the articles retrieved have been analyzed. After the first screening, records without eligibility criteria were excluded.

Remnant studies were categorized by type, according to the Oxford Centre for Evidence-Based Medicine (OCEBM).

Each study was assessed by two reviewers (R.Z., L.M.) independently and in duplicate; disagreement was resolved by the senior author (A.S). All the included studies were analyzed, and data related to topics of interest were extracted and summarized.

In detail, the data extracted included study type, mean age, site, mean follow-up, deep infections, type of graft, and type of treatment of infections. Graft infections were subclassified as early (A) and late (B) and defined as within or beyond six months of implantation [22].

Only homogeneous series that specified the number of infections for each site and/or type of graft were considered to assess cumulative data. The study is descriptive, and data are presented as total frequencies and percentages. The heterogeneity of most of the included studies did not allow any statistical analysis.

## 3. Results

**Figure 1:**
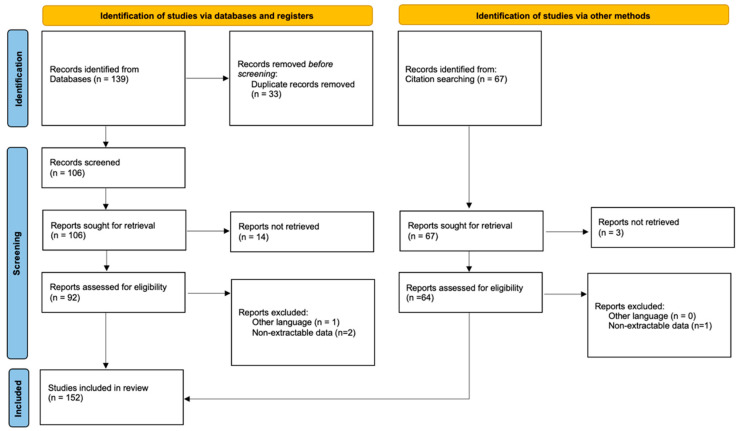
PRISMA flow diagram and the selection of studies.

A total of 139 studies were found through the electronic search, and 67 studies were added after the cross-referenced research on the bibliographies of the examined full-text articles (Figure 1).

After a preliminary analysis, a total of 152 studies reporting deep infections of graft reconstructions were included in this systematic review (16 retrospective comparative studies, 131 retrospective cohort studies, and 15 retrospective case series).

The mean age across all studies was 26.1 ± 7.3 years.

A total of 9824 grafts were retrieved. Among these, 684 (6.9%) were in the humerus (443 in the proximal segment and 220 in the diaphysis), 365 (3.7%) in the pelvis, 2041 (20.7%) in the femur (363 in the proximal segment, 940 in the diaphysis, and 738 in the distal femur), 1660 (16.8%) in the tibia (740 in the proximal tibia, 694 in the diaphysis, and 226 in the distal segment) (Table 1).

The type of graft was detailed in 9286 cases. Among these, 2481 (26.7%) were osteoarticular allografts and 2112 (22.7%) intercalary allografts. In 461 (5.0%), VFG was used in combination with recycled autografts. Recycled grafts were reported in 1573 (16.9%) of the cases and APCs in 1673 (18.0%). Four series [24,25,26,27] reported about APCs with recycled grafts (Table 2).

The mean follow-up period was 74.3 months, ranging between 10 and 198 months. However, not all the included studies reported on the duration of follow-up.

Overall, infections occurred in a total of 870 cases, with a cumulative incidence of 8.8% (Table 3).

Only a minority of the series classified the timing of infection occurrence (early in 18.4%, late in 4.8%).

Also, information regarding BGI treatment is imprecise in most of the series, being detailed in only 437 out of 870 BGI (50.2%). The most reported first treatment was debridement and implant retention (DAIR) in 187 (42.8%) cases and two-stage revision with graft removal in 152 (34.8%). Sixty-one cases (14.0%) required an amputation as the first treatment of infection. One-stage revision was attempted in only 24 (5.5%) cases, and 13 (2.9%) cases were treated with chronic suppressive antibiotics.

However, at the end of the infection treatment, only 62 (14.2%) patients were reported to have the original graft in site. One-hundred and twenty-two (27.9%) patients required an amputation; 39 (8.9%) remained with a definitive cemented spacer, whereas 224 (51.3%) received a second reconstruction (with a novel graft in 59 patients, with an APC in 68 and with a megaprosthesis in 97).

By subanalysis of homogeneous series which specified the number of infections for each site, the pelvis showed the highest incidence (20.4%, range 0–42.3%), compared to the tibia (11.0%, 0–33.3%), femur (7.3%, 0–27.8%), and humerus (3.7%, 0–9.1%).

In the pelvis subgroup, most of the cases underwent either a DAIR (43.2%) or an amputation (11.4%), compared to the higher prevalence of two-stage in the tibia (56.4%), femur (42.6%) and humerus (76.9%). However, approximately 20% of BGI in the tibia eventually required an amputation.

The subanalysis of the homogeneous series, which specified the number of infections for each type of graft, showed a cumulative incidence similar in all types of grafts: homologous 9% (range 0–33.3%), VFG 7.7% (range 0–32.0%), APC 11.0%, (range 0–42.3%), and recycled 9.3% (range 0–18.8%). The cumulative incidence of BGI was lower when homologous grafts and VFG were combined at 4.5% (range 0–25%).

Only a few data are reported on the final outcome specified by site or type of graft.

## 4. Discussion

Massive structural allografts have been most frequently used for limb reconstruction but are associated with complications such as allograft fracture, infection, and non-union because of the avascular nature of the graft.

Infection is a major cause of failure in this type of reconstruction, with many series on graft reconstructions reporting the frequency of infections. However, only a few series reported more than 10 BGI, with an infection rate ranging between 8.9–13.8%, similar to the cumulative incidence of 8.8% found in this review.

Also, the cumulative results showed a higher graft infection incidence in the pelvis and tibia, as previously reported by Beadel et al. [170], m Donati et al. [53], and Aponte-Tinao et al. [32], respectively.

A meta-analysis by Aurégan et al. [171] on APCs found that the infection rate was significantly different depending on the anatomical sites. Actually, proximal humerus APCs showed the lowest infection rate (8%) compared to the acetabulum (23%), proximal femur (10%), and proximal tibia (23%) APCs. The reasons for these differences are unknown. However, the long surgical times and poor soft tissue coverage may explain the increased infection rates for APCs of the acetabulum and proximal tibia, respectively.

Also, repeat interventions increase the risk of infection [13,172]. In the largest series published, Mankin et al. [111] described 121 infections (12.8%) in 945 patients. Of that group, however, 46 of the patients had developed infections after an additional surgery for fracture or non-union.

Aponte-Tinao et al. [32] analyzed infection rates in all types of allografts in different long bones, including intercalary, osteoarticular, and APCs, finding no differences in BGI regarding the type of graft. Our cumulative data confirmed similar BGI incidence in all types of grafts. Even though our data showed a lower BGI rate in reconstructions with combined homologous + VFG, the review by Othman et al. [173] did not find any appreciable difference in infection rates between the allograft alone and allograft/VFG groups. When used alone, the VFG is hypothesized to reduce the risk of infection secondary to its vascularity and ability to improve union. It may be that the allograft in these scenarios has an intrinsic infection risk that cannot be mitigated by the presence of an increased vascular supply, as it represents a persistent non-vascularized foreign body unable to provide a suitable immunological response.

Nevertheless, there is no general consensus on how to manage BGI, nor is there much data on the results of treatment once an infection occurs. Only a few series detailed the treatment of BGI and outcomes [32,33,53,64,67,90,102,103]. Aponte-Tinao et al. [32] observed that only 18% of the infected patients (11 out of 60) were successfully treated with surgical debridement and antibiotics without removal of the allograft. This is similar to other reports (Lord et al. [102] and Loty et al. [103], 14% each). In 49 patients (82%), the graft was removed, and a temporary cemented spacer with antibiotic was implanted to control the infection. However, only 41 (84%) were secondarily reconstructed: 24 with another bone allograft and 17 with an endoprosthesis. Of them, 14 failed with a new infection (34%), of whom 12 had an allograft (50% of the 24 patients secondarily reconstructed with an allograft), and 2 had an endoprosthesis (12% of the 17 patients with endoprosthetic reconstruction). In general, this study showed an 18% success rate of DAIR, a 45% success rate of two-stage reconstruction, and 37% of persistent infections of the initial 60 patients with BGI.

However, only a few studies provided in the literature reported on the likelihood of reinfection after treatment of BGI: in 2020, Aponte-Tinao et al., in a series of 198 allografts heterogeneous regarding the site, reported 27 BGI (13.6%). Only 6 (30%) of them were successfully treated with a DAIR, whereas 21 (78%) required graft removal in a two-stage approach; Donati et al. [53] reported 15 BGI (24.2%) in 62 proximal tibia APCs. Most of them were successfully treated with a staged approach, eventually being reconstructed with an EPR (8) or an APC (2).

Cumulative data in the literature showed a similar proportion of patients reconstructed at the end of infection treatment (51.3%).

There are several limitations to this study. Many of the included series were heterogeneous both in terms of the site and type of graft, thus affecting the incidence of infection.

There is a real lack of data on the treatment of BGI, with many series not reporting the outcome.

Furthermore, some studies had to be excluded as it was unclear whether infections were not encountered or whether they were purposely omitted, and this distinction could not be made.

## 5. Conclusions

In conclusion, this systematic review of the literature confirms a high incidence of infections in biologic reconstructions after resections of primary bone tumors. It highlights that, regardless of the type of graft, the pelvis and tibia are deemed to be at an increased risk of infections. Moreover, data from the literature suggest that, despite a DAIR being a viable attempt, in most cases, a two-stage approach with graft removal and reconstruction with endoprosthesis presented the highest chance to overcome infection, guaranteeing a reconstruction. Nevertheless, we emphasize the heterogeneity of the literature, with most of the data collected by sparse cases of graft infections in heterogeneous series, with very few details about final outcomes. Thus, future multicentric studies should focus on the management of infections after biological reconstructions in bone sarcomas.

## Figures and Tables

**Table 1:** Characteristics of included studies. NR: Not reported; EPR: endoprosthetic replacement; PH: proximal humerus; HD: humeral diaphysis; DH: distal humerus; RD: radial diaphysis; DR: distal radius; U: ulna; Pe: pelvis; PF: proximal femur; FD: femur diaphysis; DF: distal femur; PT: proximal tibia; TD: tibia diaphysis; DT: distal tibia; Fi: fibula; Fo: foot; VFG: vascularized fibula graft; N-VFG: non-vascularized fibula graft; OA: osteoarticular; APC: allograft-prosthetic composite.

Study	Patients	Age, Years (Mean)°	Type of Study	Comparison	SITE															
					PH	HD	DH	RD	DR	U	Pe	PF	FD	DF	PT	TD	DT	Fi	Fo	Others
Abdeen, 2009 [23]	36	23	retrospective	no	36	-	-	-	-		-	-	-	-	-	-	-		-	
Abed, 2009 [24]	25	19.7	retrospective	no	-	-	-	-	-		-	-	-	4	21	-	-		-	
Adam, 2020 [25]	25	10	retrospective	no	-	4	-	3	-		3	-	5	-	-	10	-		-	
Albergo, 2017 [26]	45	25	retrospective	vs. EPR	-	-	-	-	-		-	-	-	-	45	-	-		-	
Albergo, 2020 [27]	71	16	retrospective	vs. EPR	-	-	-	-	-		-	-	71	-	-	-	-		-	
Alman, 1995 [28]	26	12	retrospective	no	6	-	-	-	-		-	-	3	5	9	3	-		-	
Aponte-Tinao, 2012 [29]	83	26	retrospective	no	-	-	-	-	-		-	-	83	-	-	-	-		-	
Aponte-Tinao, 2013 [30]	70	32	retrospective	no	37	9	8	-	16		-	-	-	-	-	-	-		-	
Aponte-Tinao, 2018 [31]	22	7	retrospective	no	2	-	-	-	-		-	2	-	14	2	-	2		-	
Aponte, 2016 [32]	673	30	retrospective	no	79						-	408			186				-	
Aponte, 2020 [33]	198	22	retrospective	no	-	-	-	-	-		-	132			66				-	
Ayerza, 2016 [34]	44	27	retrospective	no	-	-	-	-	-		-	-	-	-	-	-	23		21	
Beadel, 2005 [35]	26	41	retrospective	no	-	-	-	-	-		26	-	-	-	-	-	-		-	
Bell, 1997 [36]	17	40	retrospective	no	-	-	-	-	-		17	-	-	-	-	-	-		-	
Bianchi, 2016 [37]	25	33	retrospective	no	-	-	-	-	-		-	-	-	18	7	-	-		-	
Bianchi, 2020 [38]	67	40	retrospective	no	-	-	-	-	67		-	-	-	-	-	-	-		-	
Biau, 2007 [39]	26	24	retrospective	no	-	-	-	-	-		-	-	-	-	26	-	-		-	
Biau, 2010 [40]	32	41	retrospective	no	-	-	-	-	-		-	32	-	-	-	-	-		-	
Black, 2007 [41]	6	41	retrospective	no	6	-	-	-	-		-	-	-	-	-	-	-		-	
Brunet, 2011 [42]	13	20	retrospective	no	-	-	-	-	-		-	-	8	-	-	13	-		-	
Bullens, 2009 [43]	32	27	retrospective	no	4	2	-	-	-		-	6	9	-	8	*3*	-		-	
Bus, 2014 [14]	87	17	retrospective	no	-	7	-	2	-		-	-	44	-	-	34	-		-	
Bus, 2017 [13]	38	19	retrospective	no	12	-	-	-	2		-	-	-	10	14	-	-		-	
Campanacci D, 2023 [44]	18	25	retrospective	allograft vs. allo + VFG	-	18	-	-	-		-	-	-	-	-	-	-		-	
Campanacci D, 2024 [45]	79	16	retrospective	allograft vs. allo + VFG	-	-	-	-	-		-	-	-	-	-	79	-		-	
Campanacci L, 2010 [46]	25	11	retrospective	no	-	-	-	-	-		-	-	-	13	12	-	-		-	
Campanacci L, 2015 [47]	19	11	retrospective	no	-	-	-	-	-		-	-	-	-	19	-	-		-	
Capanna, 2011 [48]	14	35	retrospective	no	-	-	-	-	-		-	-	-	-	14	-	-		-	
Chen, 2002 [49]	14	38	retrospective	no	2	-	-	-	-		-	10	-	2	-	-	-		-	
Davidson, 2005 [50]	50	23	retrospective	no		11					21		17			1				
Deijkers, 2005 [4]	35	24	retrospective	no	-	-	-	-	-		-	-	22	-	-	13	-		-	
Delloye, 2007 [51]	24	34	retrospective	no	-	-	-	-	-		24	-	-	-	-	-	-		-	
Donati, 2002 [52]	27	32	retrospective	no	-	-	-	-	-		-	27	-	-	-	-	-		-	
Donati, 2008 [53]	62	24	retrospective	no	-	-	-	-	-		-	-	-	-	62	-	-		-	
Donati, 2011 [54]	35	42	retrospective	no	-	-	-	-	-		35	-	-	-	-	-	-		-	
Dudkiewicz, 2003 [55]	11	31	retrospective	no	11	-	-	-	-		-	-	-	-	-	-	-		-	
Eid, 2011 [56]	18	39	retrospective	no	-	-	-	-	-		-	18	-	-	-	-	-		-	
El Beaino, 2019 [57]	21	41	retrospective	no	21	-	-	-	-		-	-	-	-	-	-	-		-	
Erol, 2015 [58]	18	13	retrospective	no	2	3	-	1	1		-	-	7	1	-	2	1		-	
Errani, 2019 [59]	81	13	retrospective	no	-	-	-	-	-		-	-	33	-	-	48	-		-	
Errani, 2021 [60]	5	10	retrospective	no	-	-	-	-	-		-	-	-	5	-	-	-		-	
Eward, 2010 [61]	30	29	retrospective	no	-	5	-	4	4		-	-	9	-	-	7	1		-	
Fan, 2022 [62]	9	17	retrospective	no	-	-	-	-	-		-	-	-	-	-	9	-		-	
Farfalli, 2012 [63]	26	25	retrospective	no	-	-	-	-	-		-	-	-	-	-	26	-		-	
Farfalli, 2013 [64]	86	35	retrospective	no	-	-	-	-	-		-	-	-	45	41	-	-		-	
Farid, 2006 [65]	20	44	retrospective	vs. EPR	-	-	-	-	-		-	20	-	-	-	-	-		-	
Gebert, 2006 [66]	21	15	retrospective	no	3	8	-	6	4		-	-	-	-	-	-	-		-	
Gebhardt, 1991 [67]	53	18	retrospective	no	12	-	-	-	-		-	3	2	25	10	1	-		-	
Giannini, 2020 [68]	35	22	retrospective	no												35				
Gilbert, 2009 [69]	12	34.5	retrospective	no											12					
Gorski, 2022 [70]	53	19.8	retrospective	VFG vs. N-VFG	12						-	41								
Guest, 1990 [71]	10	42	retrospective	no	-	-	-	-	-	-	10	-	-	-	-	-	-		-	
Gupta, 2017 [72]	46	32.8	retrospective	no	-	9	-	-	-	-	-	-	21	-	-	16	-		-	
Halim, 2015 [73]	12	19	retrospective	no	1		1						2	1		2	5			
Han, 2014 [74]	30	25	retrospective	no		1							20			8		1		
Han, 2015 [75]	15	19.5	retrospective	no	-	-	-	-	-	-	-	1	-	8	2	-	4	-	-	
Hanna, 2010 [76]	23	41.3	retrospective	no									23							
Hilven, 2015 [77]	74	23	retrospective	no	-	8	-	-	-	3	-	-	26	-	-	34	-	1	2	
Hones, 2023 [78]	375	NR	retrospective	no																
Hong, 2013 [79]	101	23	retrospective	no	-	17	-	1	-	-	35	-	34	-	-	14	-	-	-	
Houdek, 2016 [80]	18	11	retrospective	no	-	-	-	-	-		-	-	8	-	-	10	-		-	
Houdek, 2018 [81]	29	12	retrospective	no	-	-	-	-	-	-	-	-	15	-	-	14	-	-	-	
Humail, 2015 [82]	12	31	retrospective	no					12											
Igarashi, 2014 [83]	36	39	retrospective	no	2	1		1			7	4	4	8	5	3	1			
Ikuta, 2017 [84]	24	22	retrospective	no		4				1			17			2				
Ippolito, 2019 [85]	74	32	retrospective	no	-	16	-	-	-	-	7	-	19	-	-	25	-	-	-	-
Jager, 2010 [86]	7	12.7	retrospective	no								2	1	1	3					
Jamshidi, 2021 [87]	33	34.1	retrospective	no							15									
Jamshidi, 2023 [88]	18	10.5	retrospective	no	18															
Jeon, 2007 [89]	15	20	retrospective	no										15						
Jones, 2017 [90]	113	24	retrospective	no	19							53								
Kamalampathy, 2021 [91]	6	37	retrospective	no	-	-	6	-	-	-	-	-	-	-	-	-	-	-	-	-
Karim, 2015 [92]	14	45	retrospective	no	-	-	-	-	-	-	14	-	-	-	-	-	-	-	-	-
Kekec, 2022 [93]	19	35.8	retrospective	no	-	-	-	-	-	-	19	-	-	-	-	-	-	-	-	-
Krieg, 2007 [94]	31	29.8	retrospective	no	-	5	-	1	-	-	13	-	10	-	-	1	-	1	-	-
Krieg, 2010 [95]	18	37.3	retrospective	no							18									
Lans, 2021 [96]	33	32	retrospective	no					33											
Lenze, 2017 [97]	36	23.8	retrospective	no		6		2		1			20			5		2		
Li, 2022 [98]	26	42	retrospective	no	-	8	-	-	-	-	-	-	6	-	-	12	-	-	-	-
Li J, 2010 [8]	11	18.5	retrospective	no	-	-	-	-	-	-	-	-	5	-	-	6	-	-	-	-
Li J, 2011 [9]	8	16.5	retrospective	no	-	-	-	-	-	-	-	-	-	-	4	3	1	-	-	-
Li J, 2019 [99]	60	20.9	retrospective	no	-	10	-	-	-	-	-	-	33	-	-	17	-	-	-	-
Liu, 2012 [100]	10	27	retrospective	no	-	-	-	-	-	-	-	-	-	-	-	-	10	-	-	-
Liu, 2023 [101]	38	23	retrospective	no	3			1	-	1	-	20			13			-	-	-
Lord, 1988 [102]	283	33	retrospective	no	47			16	-	6	18	132			64			-	-	-
Loty, 1994 [103]	164	49	retrospective	no	5	3	1	-	-	-	9	69		5	-	-	-	-	-	-
Lozano, 2016 [104]	33	13	retrospective	no	-	-	-	-	-	-	-	-	-	-	32	-	-	-	-	-
Lu, 2020 [105]	23	14	retrospective	recycled vs. allograft	-	-	-	-	-	-	-	-	11	-	-	12	-	-	-	-
Lu, 2021 [106]	5	7	retrospective	no	5	-	-	-	-		-	-	-	-	-	-	-		-	
Lun, 2018 [107]	18	64	retrospective	vs. EPR	-	-	-	-	-	-	-	-	18	-	-	-	-	-	-	-
Malhotra, 2014 [108]	18	32	retrospective	no	-	-	-	-	-	-	-	18	-	-	-	-	-	-	-	-
Manfrini, 2016 [109]	47	14	retrospective	no	-	-	-	-	-	-	-	-	-	-	24	19	4	-	-	-
Mankin, 1996 [110]	718	32.2	retrospective	no																
Mankin, 2005 [111]	945	31.5	retrospective	no																
Matejovsky, 2006 [112]	72	NR	retrospective	no	15	1				1	5	2	3	21	19		3			1
McGoveran, 1997 [113]	16	51	retrospective	no								16								
Meijer, 2016 [114]	65	56	retrospective	OA vs. APC vs. EPR	65	-	-	-	-		-	-	-	-	-	-	-		-	
Miller, 2010 [115]	8	31.6	retrospective	no	1							1	1	3	2					
Min, 2015 [116]	28	35.2	retrospective	no								28								
Mo, 2013 [117]	12	29.5	retrospective	no										12						
Moran, 2006 [118]	7	10.5	retrospective	no										3	4					
Morii, 2024 [119]	707	34.5	retrospective	no	169							466								76
Muscolo, 2000 [120]	118	25	retrospective	no										73	45					
Muscolo, 2010 [121]	38	45	retrospective	no	-	-	-	-	-	-	-	38	-	-	-	-	-	-	-	-
Ogilvie, 2009 [122]	20	20	retrospective	no	4	-	-	-	1	1	-	-	-	8	6	-	-	-	-	-
Ortiz-Cruz, 1997 [123]	104	28.3	retrospective	no	19			3		2		39			38			3		
Outani, 2020 [124]	56	20	retrospective	no	13	-	-	1	-	-	1	-	-	17	22	-	-	-	1	1
Ozaki, 1996 [125]	22	27	retrospective	no	-	-	-	-	-	-	22	-	-	-	-	-	-	-	-	-
Potter, 2008 [126]	33	48.5	retrospective	no	33	-	-	-	-	-	-	-	-	-	-	-	-	-	-	-
Puerta GarciaSandoval, 2020 [127]	24	25	retrospective	no	-	-	-	-	-	-	-	-	-	-	24	-	-	-	-	-
Puri, 2012 [128]	32	15	retrospective	no	3	-	-	-	-	1	-	-	-	17	11	-	-	-	-	-
Puri, 2018 [129]	70	17	retrospective	no	-	6	-		-	1	-	-	46	-	-	17	-		-	
Rabitsch, 2013 [130]	5	42	retrospective	no	-	-	-	-	5	-	-	-	-	-	-	-	-	-	-	-
Rose, 2005 [131]	15	NR	retrospective	no	9	4	-	-	-	-	-	-	-	-	-	-	-		-	
Ruggieri, 2011 [132]	14	35	retrospective	no	14	-	-	-	-	-	-	-	-	-	-	-	-		-	
Ruiz-Moya, 2019 [133]	27	9	retrospective	no	3	-	-	1	-	-	1	16	-	-	5	-	-		1	
Sainsbury, 2014 [134]	19	10	retrospective	no	-	-	-	-	-	-	-	6	2	3	4	1	2	1	-	-
Sambri, 2020 [135]	79	19	retrospective	no													73			
Sanders, 2020 [136]	131	19	retrospective	no									89			42				
Schuh, 2014 [137]	53	20.7	retrospective	VFG vs. N-VFG	3	-	-	7	-	1	-	-	-	7	-	-	30	-	5	-
Schwarz, 2012 [138]	13	12.6	retrospective	no	-	-	-	-	-	-	-	-	-	-	4	7	2	-	-	-
Scoccianti, 2010 [139]	17	36.6	retrospective	no	-	-	-	-	17	-	-	-	-	-	-	-	-	-	-	-
Shin, 2014 [140]	6	23	retrospective	no	-	-	-	-	-	-	-	-	1	-	-	2	3	-	-	-
Song, 2012 [141]	25	NR	retrospective	vs. EPR											25					
Streitbürger, 2022 [142]	28	42.3	retrospective	no	-	-	-	-	-	-	-	-	-	17	11	-	-	-	-	-
Subhadrabandhu, 2015 [143]	22	36	retrospective	no	1	-	-	1	-	-	1	10	-	5	4	-	-	-	-	-
Sugiura, 2020 [144]	46	30.7	retrospective	no	3	-	-	-	-	1	3	-	-	21	17	-	-	-	-	1
Takenaka, 2020 [145]	33	17	retrospective	no	8	-	2	1	1	1	3	1	-	3	6	-	3	-	-	4
Takeuchi, 2023 [146]	310	27	retrospective	no	33			6			-	142			119				-	
Tan, 1997 [147]	264	31.6	retrospective	no																
Tanaka, 2012 [148]	19	19	retrospective	no	3	-	-	-	-	-	-	-	-	10	3	-	-	-	2	1
Toy, 2010 [149]	26	23	retrospective	no	-	-	-	-	-	-	-	-	-	26	-	-	-	-	-	-
Tsuchiya, 2005 [150]	28	31	retrospective	no	4	-	-	1	-	-	4	2	3	8	5	-	-	-	-	1
Van de Sande, 2010 [151]	23	44.8	retrospective	vs. EPR	23	-	-	-	-	-	-	-	-	-	-	-	-	-	-	-
van Isacker, 2011 [152]	10	32	retrospective	no	-	-	-	6	2	2	-	-	-	-	-	-	-	-	-	-
Wang, 1993 [153]	23	30	retrospective	no	2	-	-	-	-	-	-	7	-	8	6	-	-	-	-	-
Wang, 2006 [154]	20	34	retrospective	no	3	-	-	-	-	-	-	-	-	14	3	-	-	-	-	-
Wei, 2019 [155]	9	24	retrospective	vs. EPR	9	-	-	-	-	-	-	-	-	-	-	-	-	-	-	-
Weichman, 2015 [156]	12	15.8	retrospective	no	-	-	-	-	-	-	-	-	-	8	4	-	-	-	-	-
Wisanuyotin, 2022 [157]	39	22	retrospective	vs. EPR	-	-	-	-	-	-	-	-	-	21	18	-	-	-	-	-
Wisanuyotin, 2022 [158]	97	24	retrospective	auto vs. allo	4	2	-	-	4	-	-	-	1	57	-	-	29	-	-	-
Wu, 2018 [159]	164	19	retrospective	no	9	-	-	-	-	-	-	15	-	69	38	-	-	-	-	33
Yang, 2010 [160]	17	18.4	retrospective	no	-	-	-	-	-	-	-	-	-	9	8	-	-	-	-	-
Yang, 2015 [161]	58	19	retrospective	no	4	1	-	-	-	-	-	3	-	23	27	-	-	-	-	-
Yang, 2022 [162]	33	35	retrospective	no	5	-	-	-	-	1	-	-	-	10	17	-	-	-	-	-
Yao, 2020 [163]	80	19.7	retrospective	no	14	3	2	-	-	-	-	1	4	37	15	-	4	-	-	-
Yong Lee, 2017 [164]	16	35.3	retrospective	no							16									
Yong Lee, 2018 [165]	278	24	retrospective	no	34						18	137			81					8
Zaretski, 2004 [166]	30	23	retrospective	no	1	-	-	6	-	-	-	5	5	5	8	-	-		-	
Zelenski, 2013 [167]	11	10	retrospective	no	2	5	1	-	2	1	-	-	-	-	-	-	-		-	
Zhao, 2018 [168]	25	19	retrospective	no	-	-	-	-	-	-	-	-	-	-	-	-	25		-	
Zimel, 2009 [169]	38	20	retrospective	vs. EPR	-	-	-	-	-	-	-	-	-	38	-	-	-		-	

**Table 2:** Characteristics of grafts in included studies. OA: osteoarticular; IA: intercalary; N-VFG: non-vascularized fibula graft; VFG: vascularized fibula graft; RA: recycled autograft; APC: allograft-prosthetic composite; *: combination of recycled autograft in APC.

Study	Patients	Graft						
		OA	IA	N − VFG	A + VFG	VFG	RA	APC
Abdeen, 2009 [23]	36	-	-	-	-	-	-	36
Abed, 2009 [24]	25	-	-	-	25	-	-	-
Adam, 2020 [25]	25	-	-	-	-	25	-	-
Albergo, 2017 [26]	45	45		-	-	-	-	-
Albergo, 2020 [27]	71	-	71	-	-	-	-	-
Alman, 1995 [28]	26	2	24	-	-	-	-	-
Aponte-Tinao, 2012 [29]	83	-	83	-	-	-	-	-
Aponte-Tinao, 2013 [30]	70	38	9	-	-	-	-	23
Aponte-Tinao, 2018 [31]	22	6	10	-	-	-	-	-
Aponte, 2016 [32]	673	272	246	-	-	-	-	155
Aponte, 2020 [33]	198	120	78	-	-	-	-	-
Ayerza, 2016 [34]	44	16	28	-	-	-	-	-
Beadel, 2005 [35]	26	-	-	-	-	-	-	26
Bell, 1997 [36]	17	3	-	-	-	-	-	14
Bianchi, 2016 [37]	25	25	-	-	-	-	-	-
Bianchi, 2020 [38]	67	38	-	29	-	-	-	-
Biau, 2007 [39]	26	-	-	-	-	-	-	26
Biau, 2010 [40]	32	-	-	-	-	-	-	32
Black, 2007 [41]	6	-	-	-	-	-	-	6
Brunet, 2011 [42]	13	-	9	-	4	-	-	-
Bullens, 2009 [43]	32	-	14	-	-	-	-	18
Bus, 2014 [14]	87	-	87	-	-	-	-	-
Bus, 2017 [13]	38	38	-	-	-	-	-	-
Campanacci D, 2023 [44]	18	-	-	-	5	13	-	-
Campanacci D, 2024 [45]	79	-	5	-	55	19	-	-
Campanacci L, 2010 [46]	25	25	-	-	-	-	-	-
Campanacci L, 2015 [47]	19	-	-	-	-	-	-	19
Capanna, 2011 [48]	14	-	-	-	-	-	-	14
Chen, 2002 [49]	14	-	-	-	-	-	14	*
Davidson, 2005 [50]	50		16					
Deijkers, 2005 [4]	35	-	35	-	-	-	-	-
Delloye, 2007 [51]	24	24	-	-	-	-	-	-
Donati, 2002 [52]	27	-	-	-	-	-	-	27
Donati, 2008 [53]	62	-	-	-	-	-	-	62
Donati, 2011 [54]	35	-	-	-	-	-	-	35
Dudkiewicz, 2003 [55]	11	-	-	-	-	-	-	11
Eid, 2011 [56]	18	-	-	-	-	-	18	*
El Beaino, 2019 [57]	21	-	-	-	-	-	-	21
Erol, 2015 [58]	18	-	7	-	7	1	-	-
Errani, 2019 [59]	81	-	-	-	81	-	-	-
Errani, 2021 [60]	5	-	-	-	-	-	-	5
Eward, 2010 [61]	30	-	-	-	-	30	-	-
Fan, 2022 [62]	9	-	-	-	-	*	9	-
Farfalli, 2012 [63]	26	-	26	-	-	-	-	-
Farfalli, 2013 [64]	86	-	-	-	-	-	-	86
Farid, 2006 [65]	20	-	-	-	-	-	-	20
Gebert, 2006 [66]	21	-	-	-	-	21	-	-
Gebhardt, 1991 [67]	53	19	2	-	-	-	-	10
Giannini, 2020 [68]	35		19		16			
Gilbert, 2009 [69]	12							12
Gorski, 2022 [70]	53	-	-	36	-	17	-	-
Guest, 1990 [71]	10	-	-	-	-	-	-	10
Gupta, 2017 [72]	46	-	46	-	-	-	-	-
Halim, 2015 [73]	12				12			
Han, 2014 [74]	30		17				13	
Han, 2015 [75]	15	-	15	-	-	-	-	-
Hanna, 2010 [76]	23							
Hilven, 2015 [77]	74	-	-	-	-	74	-	-
Hones, 2023 [78]	375							
Hong, 2013 [79]	101	-	-	-	-	-	101	-
Houdek, 2016 [80]	18				18			
Houdek, 2018 [81]	29	-	11	-	18	-	-	-
Humail, 2015 [82]	12			12				
Igarashi, 2014 [83]	36	16	13					7
Ikuta, 2017 [84]	24						24	
Ippolito, 2019 [85]	74	15	38	-	-	-	-	21
Jager, 2010 [86]	7				7			
Jamshidi, 2021 [87]	33		15					
Jamshidi, 2023 [88]	18		18					
Jeon, 2007 [89]	15						15	*
Jones, 2017 [90]	113	-	28	-	15	32	-	38
Kamalampathy, 2021 [91]	6	6	-	-	-	-	-	-
Karim, 2015 [92]	14	-	6	-	-	4	-	4
Kekec, 2022 [93]	19	-	19	-	-	-	-	-
Krieg, 2007 [94]	31	-	-	31	-	-	-	-
Krieg, 2010 [95]	18			18				
Lans, 2021 [96]	33	33						
Lenze, 2017 [97]	36			36				
Li, 2022 [98]	26	-	-	-	-	26	-	-
Li J, 2010 [8]	11	-	-	-	11	-	-	-
Li J, 2011 [9]	8	-	-	-	8	-	-	-
Li J, 2019 [99]	60	-	-	-	60	-	-	-
Lord, 1988 [102]	283	188	47	-	-	-	-	48
Loty, 1994 [103]	164	-	-	-	-	-	-	164
Lozano, 2016 [104]	33	23	9	-	-	-	-	1
Lu, 2020 [105]	23	-	-	-	15	-	8	-
Lu, 2021 [106]	5	-	-	-	5	-	-	-
Lun, 2018 [107]	18	-	18	-	-	-	-	-
Malhotra, 2014 [108]	18	-	-	-	-	-	-	18
Manfrini, 2016 [109]	47	-	-	-	47	-	-	-
Mankin, 1996 [110]	718	386	163	-	-	-	-	169
Mankin, 2005 [111]	945	483	282					174
Matejovsky, 2006 [112]	72	23	28			20		
McGoveran, 1997 [113]	16							16
Meijer, 2016 [114]	65	45	-	-	-	-	-	20
Miller, 2010 [115]	8	5	3					1
Min, 2015 [116]	28							28
Mo, 2013 [117]	12							12
Moran, 2006 [118]	7					7		
Morii, 2024 [119]	707					140	505	
Muscolo, 2000 [120]	118	118						
Muscolo, 2010 [121]	38	-	-	-	-	-	-	38
Ogilvie, 2009 [122]	20	20	-	-	-	-	-	-
Ortiz-Cruz, 1997 [123]	104		104					
Outani, 2020 [124]	56	24	22	-	-	-	-	10
Ozaki, 1996 [125]	22	-	13	-	-	-	-	9
Potter, 2008 [126]	33	17	16	-	-	-	-	-
Puerta GarciaSandoval, 2020 [127]	24	-	-	-	-	-	-	24
Puri, 2012 [128]	32	-	-		-	-	32	-
Puri, 2018 [129]	70	-	-	-	-	-	70	-
Rabitsch, 2013 [130]	5	5	-	-	-	-	-	-
Rose, 2005 [131]	15	1	4	-	-	9	-	-
Ruggieri, 2011 [132]	14	-	-	-	-	-	-	14
Ruiz-Moya, 2019 [133]	27	-	-	-	-	27	-	-
Sainsbury, 2014 [134]	19	-	-	-	-	19	-	-
Sambri, 2020 [135]	79	11	17					45
Sanders, 2020 [136]	131		131					
Schuh, 2014 [137]	53	-	-	27	-	26	-	-
Schwarz, 2012 [138]	13	-	-	-	-	13	-	-
Scoccianti, 2010 [139]	17	17	-	-	-	-	-	-
Shin, 2014 [140]	6	-	5	-	-	-	-	1
Song, 2012 [141]	25							25
Streitbürger, 2022 [142]	28	-	-	-	-	-	-	28
Subhadrabandhu, 2015 [143]	22	-	-	-	-	-	-	22
Sugiura, 2020 [144]	46	-	-	0	-	-	46	-
Takenaka, 2020 [145]	33	33	-	-	-	-	-	-
Takeuchi, 2023 [146]	310	-		-	-	-	310	*
Tan, 1997 [147]	264	176	45					43
Tanaka, 2012 [148]	19	-	-	-	-	19	-	-
Toy, 2010 [149]	26	26	-	-	-	-	-	-
Tsuchiya, 2005 [150]	28	-	-	28	-	-	-	-
Van de Sande, 2010 [151]	23	13	-	-	-	-	-	10
van Isacker, 2011 [152]	10	5	5	-	-	-	-	-
Wang, 1993 [153]	23	12	5	-	-	-	-	6
Wang, 2006 [154]	20	20	-	-	-	-	-	-
Wei, 2019 [155]	9	-	-	-	-	-	-	9
Weichman, 2015 [156]	12	-	-	-	12	-	-	-
Wisanuyotin, 2022 [157]	39	39	-	-	-	-	-	-
Wisanuyotin, 2022 [158]	97	-	47	50	-	-	-	-
Wu, 2018 [159]	164	-	85		-	-	79	-
Yang, 2010 [160]	17	-	-	-	17	-	-	-
Yang, 2015 [161]	58	-	-	58	-	-	-	-
Yang, 2022 [162]	33	-	-	33	-	-	-	-
Yao, 2020 [163]	80	-	52	28	-	-	-	-
Yong Lee, 2017 [164]	16						16	
Yong Lee, 2018 [165]	278						253	
Zaretski, 2004 [166]	30	-	-	1	-	29	-	-
Zelenski, 2013 [167]	11	-	-	-	-	11	-	-
Zhao, 2018 [168]	25	6	-	14	5	-	-	-
Zimel, 2009 [169]	38	38	-	-	-	-	-	-

**Table 3:** Characteristics of graft infections and their treatment. DAIR: debridement and implant retention; APC: allograft-prosthetic composite.

Study	Patients	Infections (n)	Infections (%)	Follow-Up, Months (mean)	Type of Infection		Treatment					Final Treatment						Graft Failure	PJI Recurrence (%)
					Early	Late	Dair	One Stage	Two Stage	Antibiotic	Amputation	Amputation	Allograft	APC	Prosthesis	Arthrodesis	Cement Spacer		
Abdeen, 2009 [23]	36	0	0.0	60	-	-	-	-	-	-	-	-	-	-	-	-	-	0	-
Abed, 2009 [24]	25	1	4.0	140	1	-	-	-	-	-	1	1	-	-	-	-	-	0	-
Adam, 2020 [25]	25	8	32.0	86	-	-	-	-	-	-	-	-	-	-	-	-	-	0	
Albergo, 2017 [26]	45	9	20.0	89	9	-	-	-	5	-	1	1	-	3	2	3	-	5	-
Albergo, 2020 [27]	71	1	1.4	129	1	-	-	-	1	-	-	-	-	-	1	-	-	1	.
Alman, 1995 [28]	26	3	11.5	63	-	-	-	-	1	-	2	2	-	-	-	-	1	3	-
Aponte-Tinao, 2012 [29]	83	1	1.2	61	1	-	-	-	1	-	-	-	1	-	-	-	-	1	-
Aponte-Tinao, 2013 [30]	70	2	2.9	60	2	-	-	-	2	-	-	-	1	-	1	-	-	2	-
Aponte-Tinao, 2018 [31]	22	1	4.5	162	1	-	-	-	1	-	-	-	1	-	-	-	-	1	-
Aponte, 2016 [32]	673	60	8.9	106	-	-	60	-	49 (all after DAIR)	-	-	4	13	11	17	-	4	49	14
Aponte, 2020 [33]	198	27	13.6	192	20	7	6	-	21	-	-	1	7	6	7	-	-	21	3
Ayerza, 2016 [34]	44	1	2.3	53	1	-	-	-	1	-	-	-	1	-	-	-	-	1	-
Beadel, 2005 [35]	26	11	42.3	55	-	-	-	-	-	-	-	-	-	-	-	-	-	7	-
Bell, 1997 [36]	17	2	11.8	84	2	-	-	-	1	-	1	1	-	-	-	-	-	2	-
Bianchi, 2016 [37]	25	0	0.0	123	-	-	-	-	-	-	-	-	-	-	-	-	-	-	-
Bianchi, 2020 [38]	67	0	0.0	105	-	-	-	-	-	-	-	-	-	-	-	-	-	-	-
Biau, 2007 [39]	26	6	23.1	126	6	-	-	-	5	-	1	1	-	-	3	2	-	6	-
Biau, 2010 [40]	32	4	12.5	68	-	-	-	1	3	-	-	-	-	1	3	-	-	4	1
Black, 2007 [41]	6	0	0.0	25	-	-	-	-	-	-	-	-	-	-	-	-	-	-	-
Brunet, 2011 [42]	13	3	23.1	48	2	1	3	-	-	-	-	-	-	-	-	-	-	0	
Bullens, 2009 [43]	32	5	15.6	63	5	-	-	1	-	3	1	1	-	-	1	-	-	2	-
Bus, 2014 [14]	87	12	13.8	84	-	-	-	-	-	-	-	-	-	-	-	-	-	-	-
Bus, 2017 [13]	38	3	7.9	20	-	-	-	-	-	-	-	-	-	-	-	-	-	-	-
Campanacci D, 2023 [44]	18	0	0.0	176	-	-	-	-	-	-	-	-	-	-	-	-	-	-	-
Campanacci D, 2024 [45]	79	6	7.6	148	-	-	6	-	-	-	-	1	-	-	-	-	-	2	-
Campanacci L, 2010 [46]	25	0	0.0	124	-	-	-	-	-	-	-	-	-	-	-	-	-	-	-
Campanacci L, 2015 [47]	19	1	5.3	78	1	-	-	-	-	-	1	1	-	-	-	-	-	1	-
Capanna, 2011 [48]	14	2	14.3	54	-	-	-	-	2	-	-	-	-	-	1	1	-	2	-
Chen, 2002 [49]	14	0	0.0	43	-	-	-	-	-	-	-	-	-	-	-	-	-	-	-
Davidson, 2005 [50]	50	0	0.0	38															
Deijkers, 2005 [4]	35	3	8.6	86	-	-	1	-	2	-	-	-	1	-	-	-	-	2	0
Delloye, 2007 [51]	24	3	12.5	41	-	-	-	-	-	-	-	-	-	-	-	-	-	-	-
Donati, 2002 [52]	27	1	3.7	58	1	-	-	-	-	-	-	-	-	-	1	-	-	1	-
Donati, 2008 [53]	62	15	24.2	72	-	-	3	-	10	-	2	2	-	2	8	-	-	12	-
Donati, 2011 [54]	35	8	22.9	120	-	-	-	-	-	-	3	3	-	-	3	-	1	-	-
Dudkiewicz, 2003 [55]	11	1	9.1	68	1	-	-	-	-	1	-	-	-	-	-	-	-	0	-
Eid, 2011 [56]	18	2	11.1	93	1	1	2	-	-	-	-	-	-	-	-	-	-	2	2
El Beaino, 2019 [57]	21	1	4.8	97	-	-	-	-	1	-	-	-	-	-	1	-	-	1	0
Erol, 2015 [58]	18	1	5.6	46	1	-	1	-	-	-	-	-	-	-	-	-	-	0	-
Errani, 2019 [59]	81	5	6.2	96			1	-	4	-	-	-	-	-	-	-	-	4	-
Errani, 2021 [60]	5	1	20.0	70	-	1	-	-	1	-	-	-	-	-	1	-	-	1	
Eward, 2010 [61]	30	3	10.0	59	-	3	1	-	1	-	1	1	-	-	-	-	-	2	-
Fan, 2022 [62]	9	0	0.0	49	-	-	-	-	-	-	-	-	-	-	-	-	-	-	-
Farfalli, 2012 [63]	26	3	11.5	73	3	-	-	-	3	-	-	-	2	-	-	-	1	3	0
Farfalli, 2013 [64]	86	11	12.8	72	-	-	-	-	9	-	2	4	-	1	4	2	-	11	-
Farid, 2006 [65]	20	1	5.0	76	-	1	-	-	1	-	-	-	-	-	1	-	-	1	-
Gebert, 2006 [66]	21	1	4.8	44	1	-	1	-	-	-	-	-	-	-	-	-	-	0	
Gebhardt, 1991 [67]	53	16	30.2	25	-	1	5	1	7	2	1	7	4	-	-	1	2	14	-
Giannini, 2020 [68]	35	4	11.4	36	4	-	1	-	1	-	2	2	-	-	-	-	-		
Gilbert, 2009 [69]	12	1	8.3	49	1	-	1	-	-	-	-	-	-	1	-	-	-	-	
Gorski, 2022 [70]	53	4	7.5	178.8	-	-	-	-	-	-	-	-	-	-	-	-	-		
Guest, 1990 [71]	10	1	10.0	25	-	-	-	-	-	-	1	1	-	-	-	-	-	-	-
Gupta, 2017 [72]	46	4	8.7	92	-	-	1	-	2	1	-	-	1	-	1	-	1	1	25%
Halim, 2015 [73]	12	1	8.3	63	1				1										
Han, 2014 [74]	30	3	10.0	79		3			3										
Han, 2015 [75]	15	0	0.0	63.2	-	-	-	-	-	-	-	-	-	-	-	-	-	-	-
Hanna, 2010 [76]	23	1	4.3		1				1						1			1	0
Hilven, 2015 [77]	74	3	4.1	77	-	-	-	-	-	-	-	-	-	-	-	-	-	-	-
Hones, 2023 [78]	375	9	2.4	NR															
Hong, 2013 [79]	101	0	0.0	52.8	-	-	-	-	-	-	-	-	-	-	-	-	-	-	-
Houdek, 2016 [80]	18	0	0.0	96	-	-	-	-	-	-	-	-	-	-	-	-	-	-	-
Houdek, 2018 [81]	29	2	6.9	156	-	-	-	-	1	-	1	1	-	-	-	-	-	-	-
Humail, 2015 [82]	12	0	0.0	24															
Igarashi, 2014 [83]	36	4	11.1	101	3		3		1										
Ikuta, 2017 [84]	24	0	0.0	88		3	1		1		1	1							
Ippolito, 2019 [85]	74	5	6.8	105	-	5	-	-	4	-	1	-	-	-	4	-	-		10% (APC) 7% (Ostart Allo)
Jager, 2010 [86]	7	1	14.3	44															
Jamshidi, 2021 [87]	33	0	0.0	80															
Jamshidi, 2023 [88]	18	1	5.6	88	1				1				1						
Jeon, 2007 [89]	15	0	0.0	56															
Jones, 2017 [90]	113	10	8.8	80.3	-	-	10	-	-	-	-	-	-	-	-	-	-		
Kamalampathy, 2021 [91]	6	0	0.0	69.5	-	-	-	-	-	-	-	-	-	-	-	-	-		
Karim, 2015 [92]	14	2	14.3	19	-	-	2	-	-	-	-	-	-	-	-	-	-		
Kekec, 2022 [93]	19	6	31.6	97			6	-	-	-	-	-	-	-	-	-	-		
Krieg, 2007 [94]	31	1	3.2	5.6			-	-	1	-	-								
Krieg, 2010 [95]	18	0	0.0	121															
Lans, 2021 [96]	33	0	0.0	156															
Lenze, 2017 [97]	36	1	2.8	100															
Li, 2022 [98]	26	1	3.8	73	-	-	1	-	-	-	-	-	-	-	-	-	-	-	
Li J, 2010 [8]	11	0	0.0	34.1	-	-	-	-	-	-	-	-	-	-	-	-	-		
Li J, 2011 [9]	8	0	0.0	38.4	-	-	-	-	-	-	-	-	-	-	-	-	-		
Li J, 2019 [99]	60	2	3.3	52.3	2	-	1	1	-	-	-	1	-	-	-	-	-		
Liu, 2012 [100]	10	0	0.0	81	-	-	-	-	-	-	-	-	-	-	-	-	-	-	
Liu, 2023 [101]	38	0	0.0	57	-	-	-	-	-	-	-	-	-	-	-	-	-	-	
Lord, 1988 [102]	283	33	11.7	71	33	-	6	16	11	-	-	27	6	-	-	-	-	27	
Loty, 1994 [103]	164	18	11.0	nr	-	-	7	1	7	-	1	1	4	-	2	-	11		
Lozano, 2016 [104]	33	5	15.2	55	-	-	-	-	4	-	1	1	2	-	2	-	-		
Lu, 2020 [105]	23	0	0.0	45	-	-	-	-	-	-	-	-	-	-	-	-	-		
Lu, 2021 [106]	5	0	0.0	47	-	-	-	-	-	-	-	-	-	-	-	-	-	-	-
Lun, 2018 [107]	18	5	27.8	10	-	-	-	-	-	-	-	-	-	-	-	-	-		
Malhotra, 2014 [108]	18	0	0.0	54	-	-	-	-	-	-	-	-	-	-	-	-	-		
Manfrini, 2016 [109]	47	2	4.3	84	-	-	1	-	2	-	-	-	-	-	1	-	1		
Mankin, 1996 [110]	718	82	11.4	67	-	-	-	-	-	-	-	-	-	-	-	-	-		
Mankin, 2005 [111]	945	75	7.9	84	-	-					22	22							
Matejovsky, 2006 [112]	72	7	9.7	-	6	1													
McGoveran, 1997 [113]	16	3	18.8	47					2	1					1		1		
Meijer, 2016 [114]	65	5	7.7	28	1	4	-	-	5	-	-	-	-	-	-	-	-	5	0
Miller, 2010 [115]	8	0	0.0	18.1															
Min, 2015 [116]	28	0	0.0	56															
Mo, 2013 [117]	12	1	8.3	12	1	-	-	-	1	-	-	-	-	1	-	-	-		
Moran, 2006 [118]	7	0	0.0	36	-	-	-	-	-	-	-	-	-	-	-	-	-		
Morii, 2024 [119]	707	76	10.7	44	-	-	-	-	-	-	-	-	-	-	-	-	-		
Muscolo, 2000 [120]	118	13	11.0	66	-	-	-	-	-	-	1	1	1	3	2	5	1		
Muscolo, 2010 [121]	38	3	7.9	36	-	-	1	-	-	-	-	-	-	-	2	-	1	-	-
Ogilvie, 2009 [122]	20	2	10.0	120	-	-	-	1	-	-	1	1	-	-	1	-	-	-	-
Ortiz-Cruz, 1997 [123]	104	12	11.5	67.2	12	-	-	-	-	-	6	6	-	-	-	-	-		
Outani, 2020 [124]	56	7	12.5	198	-	-	-	-	-	-	-	-	-	-	-	-	-	-	-
Ozaki, 1996 [125]	22	8	36.4	48	8	-	8	-	-	-	-	2	3	1	2	-	-	-	1
Potter, 2008 [126]	33	1	3.0	98	-	-	-	-	-	-	-	-	-	-	-	-	-	-	-
Puerta GarciaSandoval, 2020 [127]	24	1	4.2	132	1	-	-	-	-	-	1	1	-	-	-	-	-	-	-
Puri, 2012 [128]	32	0	0.0	34	-	-	-	-	-	-	-	-	-	-	-	-	-	-	-
Puri, 2018 [129]	70	8	11.4	61	4	4	3	-	5	-	-	-	-	-	-	-	-	5	-
Rabitsch, 2013 [130]	5	0	0.0	32	-	-	-	-	-	-	-	-	-	-	-	-	-	-	-
Rose, 2005 [131]	15	5	33.3	68	-	-	5	-	-	-	-	-	-	-	-	-	-	0	-
Ruggieri, 2011 [132]	14	1	7.1	25	-	1	-	-	1	-	-	-	-	-	1	-	-	-	-
Ruiz-Moya, 2019 [133]	27	0	0.0	44															
Sainsbury, 2014 [134]	19	0	0.0	57	-	-	-	-	-	-	-	-	-	-	-	-	-	-	-
Sambri, 2020 [135]	79	5	6.3	77			2	-	2	-	1	1	-	2	-	-	-		
Sanders, 2020 [136]	131	8	6.1	168	5					2								6	
Schuh, 2014 [137]	53	0	0.0	52	-	-	-	-	-	-	-	-	-	-	-	-	-	-	-
Schwarz, 2012 [138]	13	0	0.2	63	-	-	-	-	-	-	-	-	-	-	-	-	-	-	-
Scoccianti, 2010 [139]	17	0	0.0	58.9	-	-	-	-	-	-	-	-	-	-	-	-	-	-	-
Shin, 2014 [140]	6	1	16.7	26	-	-	1	-	-	-	-	-	-	-	-	-	-	-	-
Song, 2012 [141]	25	5	20.0	82	5		3	2								5		5	
Streitbürger, 2022 [142]	28	3	10.7	75	-	-	-	-	1	-	1	1	-	-	-	-	-	-	-
Subhadrabandhu, 2015 [143]	22	2	9.1	63	-	-	-	-	-	-	-	-	-	22	-	-	-	-	-
Sugiura, 2020 [144]	46	6	13.0	103	-	-	4	-	-	-	-	-	-	-	-	-	-		
Takenaka, 2020 [145]	33	4	12.1	125	-	-	-	-	2	-	-	-	-	-	2	-	1	-	-
Takeuchi, 2023 [146]	310	34	11.0	92	-	-	-	-	-	-	-	-	-	-	-	-	-	20	-
Tan, 1997 [147]	264	20	7,6	87.6															
Tanaka, 2012 [148]	19	3	15.8	84	-	-	-	-	-	-	-	-	-	-	-	-	-	-	-
Toy, 2010 [149]	26	4	15.4	56.6	-	-	4	-	-	-	-	-	-	1	1	-	-	-	-
Tsuchiya, 2005 [150]	28	3	10.7	28	-	-	1	-	-	-	-	-	-	-	-	-	-	-	-
Van de Sande, 2010 [151]	23	2	8.7	120	-	-	-	-	-	-	-	-	-	-	-	-	-	-	-
van Isacker, 2011 [152]	10	0	0.0	110	-	-	-	-	-	-	-	-	-	-	-	-	-	-	-
Wang, 1993 [153]	23	2	8.7	48	-	2	-	-	-	-	-	-	-	-	-	-	-	-	-
Wang, 2006 [154]	20	6	30.0	160	-	-	-	-	6	-	-	1	-	-	5	-	-	-	-
Wei, 2019 [155]	9	0	0.0	40	-		-	-	-	-	-	-	-	9	-	-	-	-	-
Weichman, 2015 [156]	12	2	16.7	41	-	-	-	-	-	-	-	-	-	-	-	-	-	-	-
Wisanuyotin, 2022 [157]	39	4	10.3	70	-	-	2	-	2	-	-	-	2	-	-	2	-	-	-
Wisanuyotin, 2022 [158]	97	6	6.2	86	-	-	6	-	-	-	-	-	-	-	-	-	-	-	-
Wu, 2018 [159]	164	1	0.6	75	6	4	2	-	-	-	2	2	-	-	-	-	2	-	-
Yang, 2010 [160]	17	0	0.0	20.2	-	-	-	-	-	-	-	-	-	-	-	-	-	-	-
Yang, 2015 [161]	58	8	13.8	54	-	-	8	-	-	-	-	-	-	-	-	-	-	-	-
Yang, 2022 [162]	33	1	3.0	50	-	-	-	-	-	-	1	1	-	-	-	-	-	-	-
Yao, 2020 [163]	80	4	5.0	42	-	-	-	-	-	-	-	-	-	-	-	-	-	-	-
Yong Lee, 2017 [164]	16	3	18.8	13			3												
Yong Lee, 2018 [165]	278	33	11.9	113			-	-	-	-	-	-	-	-	-	-	-		
Zaretski, 2004 [166]	30	3	10.0	30	-	-	-	-	-	3	-	-	-	-	-	-	-	-	-
Zelenski, 2013 [167]	11	0	0.0	57	-	-	-	-	-	-	-	-	-	-	-	-	-	-	-
Zhao, 2018 [168]	25	3	12.0	60	3	-	1	-	-	-	-	1	-	-	-	-	1	-	-
Zimel, 2009 [169]	38	7	18.4	24	-	-						6	8	-	9	-	-	-	47%

## Data Availability

The data presented in this study are available on request from the corresponding author.
